# Synthesis and crystal structure of diiso­thio­cyanato­tetra­kis­(4-methyl­pyridine *N*-oxide)cobalt(II) and diiso­thio­cyanato­tris­(4-methyl­pyridine *N*-oxide)cobalt(II) showing two different metal coordination polyhedra

**DOI:** 10.1107/S2056989024000471

**Published:** 2024-01-26

**Authors:** Christian Näther, Inke Jess

**Affiliations:** a Institut für Anorganische Chemie, Universität Kiel, Germany; University of Aberdeen, United Kingdom

**Keywords:** synthesis, coordination compound, cobalt thio­cyanate, 4-methyl­pyridine *N*-oxide, crystal structure

## Abstract

The reaction of Co(NCS)_2_ with 4-methyl­pyridine *N*-oxide (C_6_H_7_NO) leads to the formation of two compounds, namely, tetra­kis­(4-methyl­pyridine *N*-oxide-κ*O*)bis­(thio­cyanato-κ*N*)cobalt(II), [Co(NCS)_2_(C_6_H_7_NO)_4_] (**1**), and tris­(4-methyl­pyridine *N*-oxide-κ*O*)bis­(thio­cyanato-κ*N*)cobalt(II), [Co(NCS)_2_(C_6_H_7_NO)_3_] (**2**). The asymmetric unit of **1** consists of one Co^II^ cation located on a centre of inversion, as well as one thio­cyanate anion and two 4-methyl­pyridine *N*-oxide coligands in general positions. The Co^II^ cations are octa­hedrally coordinated by two terminal *N*-bonding thio­cyanate anions in *trans* positions and four 4-methyl­pyridine *N*-oxide ligands. In the extended structure, these complexes are linked by C—H⋯O and C—H⋯S inter­actions. In compound **2**, two crystallographically independent complexes are present, which occupy general positions. In each of these complexes, the Co^II^ cations are coordinated in a trigonal–bipyramidal manner by two terminal *N*-bonding thio­cyanate anions in axial positions and by three 4-methyl­pyridine *N*-oxide ligands in equatorial positions. In the crystal, these complex mol­ecules are linked by C—H⋯S inter­actions. For compound **2**, a nonmerohedral twin refinement was performed. Powder X-ray diffraction (PXRD) reveals that **2** was nearly obtained as a pure phase, which is not possible for compound **1**. Differential thermoanalysis and thermogravimetry data (DTA–TG) show that compound **2** start to decompose at about 518 K.

## Abstract 2

In the crystal structure of the title compounds, the Co^II^ cations are either sixfold or fivefold coordinated by two thio­cyanate anions and four or three 4-methyl­pyridine *N*-oxide coligands within a slightly distorted octa­hedral or a trigonal–bipyramidal coordination polyhedron.

## 1. Chemical context

Complexes based on transition-metal thio­cyanates are an important class of compounds in coordination chemistry. Because of their versatile coordination behaviour they show a variety of coordination modes and a large structural variability, which can lead to networks of different dimensionality (Buckingham, 1994 ▸; Kabešová *et al.*, 1995 ▸; Barnett *et al.*, 2002 ▸; Werner *et al.*, 2014 ▸; Neumann *et al.*, 2018 ▸). In this context, compounds based on paramagnetic metal cations are of special inter­est, because they show very versatile magnetic behaviour. We have been inter­ested in the structural, thermal and magnetic behaviour of thio­cyanate compounds with 3*d*-metal cations for several years. In terms of magnetic properties, compounds based on Co^II^, in which the cations are linked into chains, are of special inter­est, because they show a variety of magnetic properties, including ferro- or single-chain magnetism (Mautner *et al.*, 2018*b* ▸; Rams *et al.*, 2017 ▸, 2020 ▸, 2023 ▸; Wöhlert *et al.*, 2013 ▸). 
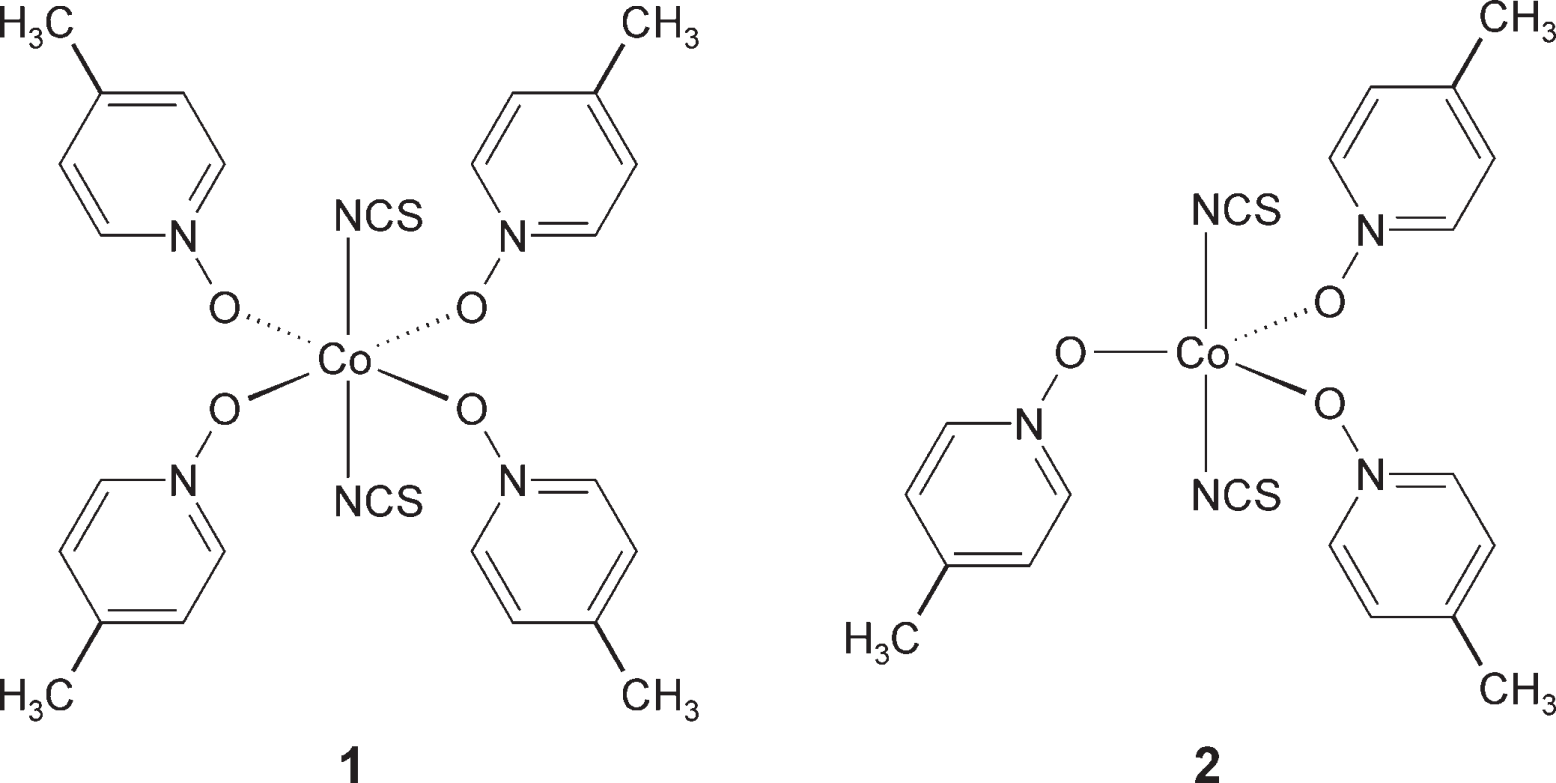


Concerning the coordination behaviour of cobalt thio­cyanates, in the majority of compounds the Co^II^ cations are sixfold coordinated within a slightly distorted octa­hedral geometry and more than 1000 such structures can be found in the Cambridge Structural Database (CSD; Version 5.43, last update March 2023; Groom *et al.*, 2016 ▸). Depending on the nature of the coligand, in some cases, the Co^II^ cations are tetra­hedrally coordinated and about 280 of such structures are reported in the CSD. In very rare cases, a compound with an octa­hedral coordination and another compound with a tetra­hedral coordination were obtained in a synthesis using the same coligand (Mautner *et al.*, 2018*b* ▸). In contrast, Co(NCS)_2_ compounds with a fivefold coordination are uncommon and only about 60 structures have been reported. In this context, it is noted that we have reported the first Co^II^ chain compound, in which the Co^II^ cations shows an alternating five- and sixfold coordination (Böhme *et al.*, 2022 ▸).

In our recent work, however, we exclusively used *N*-donor coligands, such as pyridine derivatives, for the synthesis of new thio­cyanate coordination polymers, but in the course of our systematic work we started to use also *O*- or *S*-donor coligands (Jochim *et al.*, 2020 ▸). For *O*-donor coligands, pyridine *N*-oxide derivatives might be suitable, for which only 11 compounds with cobalt are reported in the CSD (see *Database survey* section). In this context, it is noted that we recently reported on new compounds with the composition Co(NCS)_2_(2-methyl­pyridine *N*-oxide) and Co(NCS)_2_(3-cyano­pyridine *N*-oxide)_4_. In the first compound, the cations are octa­hedrally coordinated by two *O*-bonding 2-methyl­pyridine *N*-oxide ligands, as well as by two thio­cyanate anions, and are con­nected by μ-1,3(*N*,*S*)-bridging thio­cyanate anions into chains that are further linked into layers by pairs of μ-1,1(*O*,*O*) bridging coligands (Näther & Jess, 2024 ▸). In contrast, the second compound consists of discrete octa­hedral complexes (Näther & Jess, 2023 ▸). In continuation of this work, we tried to prepare similar compounds with 4-methyl­pyridine *N*-oxide (C_6_H_7_NO), for which only one compound with the composition Co(NCS)_2_(C_6_H_7_NO)(methanol) is reported, in which the Co^II^ cations are also sixfold coordinated by two O atoms of the coligand, one methanol mol­ecule, as well as by one terminal and two bridging thio­cyanate anions, and linked into chains by alternating pairs of thio­cyanate anions and 4-methyl­pyridine *N*-oxide coligands (CSD refcode REKBUF; Shi *et al.*, 2006*a* ▸). Within our synthetic work, crystals of two different crystalline phases were obtained. Single-crystal structure analysis shows that discrete complexes had formed, in which the Co^II^ cations shows either a sixfold or a fivefold coordination. We note that some transition-metal thio­cyanate compounds with pyridine *N*-oxide derivatives are reported in the literature that also form discrete complexes, but in none of them are the cations fivefold coordinated (see *Database survey* section).

## 2. Structural commentary

**Figure 1:**
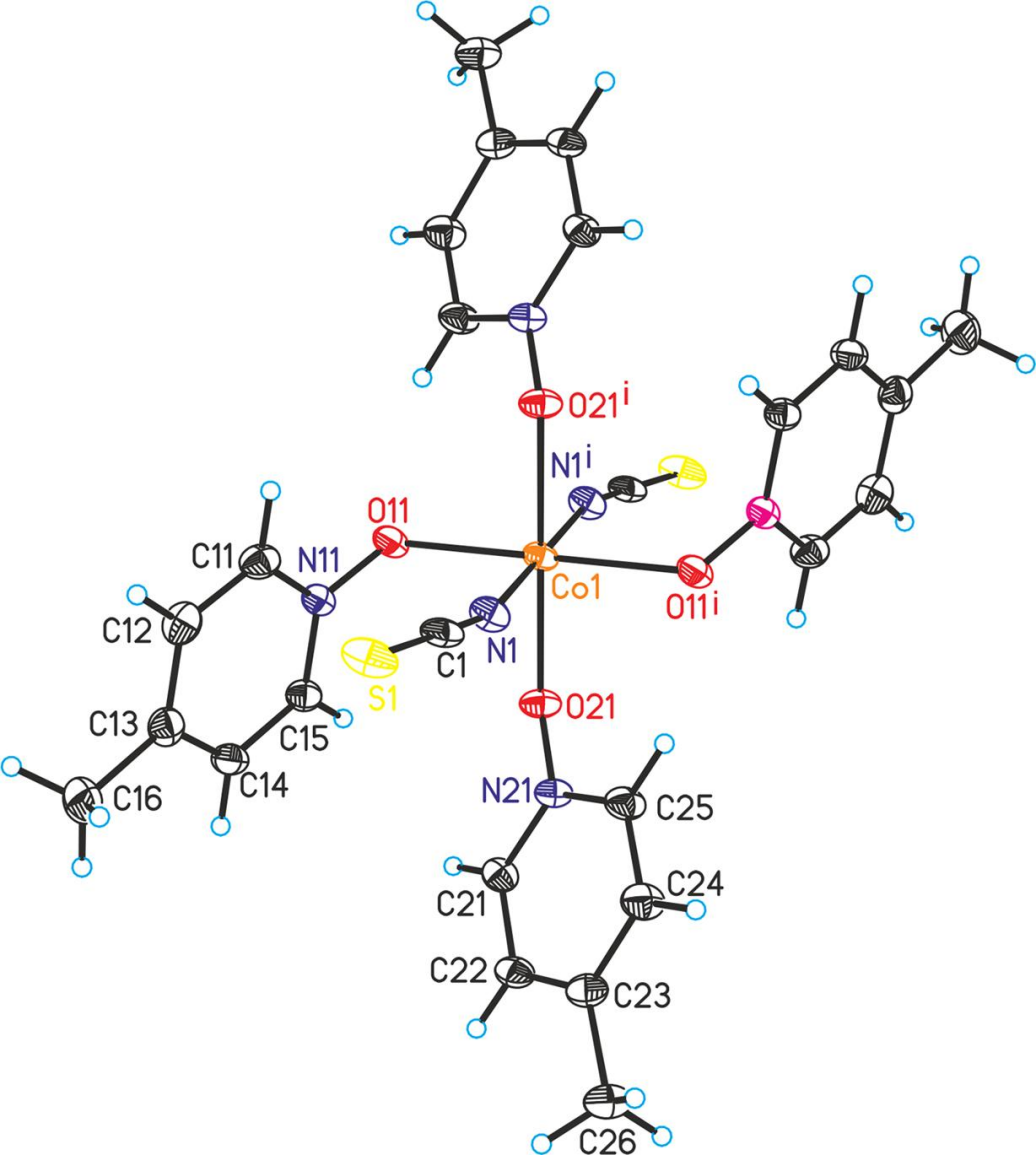
Crystal structure of compound **1**, with displacement ellipsoids drawn at the 50% probability level. [Symmetry code: (i) −*x* + 1, −*y*, −*z* + 1.]

The reactions of different molar ratios of Co(NCS)_2_ and 4-methyl­pyridine *N*-oxide leads to the formation of crystals of two compounds with the compositions Co(NCS)_2_(C_6_H_7_NO)_4_ (**1**) and Co(NCS)_2_(C_6_H_7_NO)_3_ (**2**). Compound **2** can be prepared in larger amounts, whereas a few crystals of compound **1** were accidently obtained in only one batch (see *Synthesis and crystallization* section). The asymmetric unit of compound **1** consists of one Co^II^ cation located on a crystallographic centre of inversion, as well as one thio­cyanate anion and two 4-methyl­pyridine *N*-oxide coligands in general positions (Fig. 1 ▸). The cations are sixfold coordinated by two terminal *N*-bonded thio­cyanate anions in *trans* positions and by four O atoms of the 4-methyl­pyridine *N*-oxide coligands. From the bond lengths and angles it is apparent that the *trans*-CoN_2_O_4_ octa­hedra are slightly distorted (Table 1 ▸). It is noted that numerous similar complexes with a distorted octa­hedral coordination are reported in the literature.

**Figure 2:**
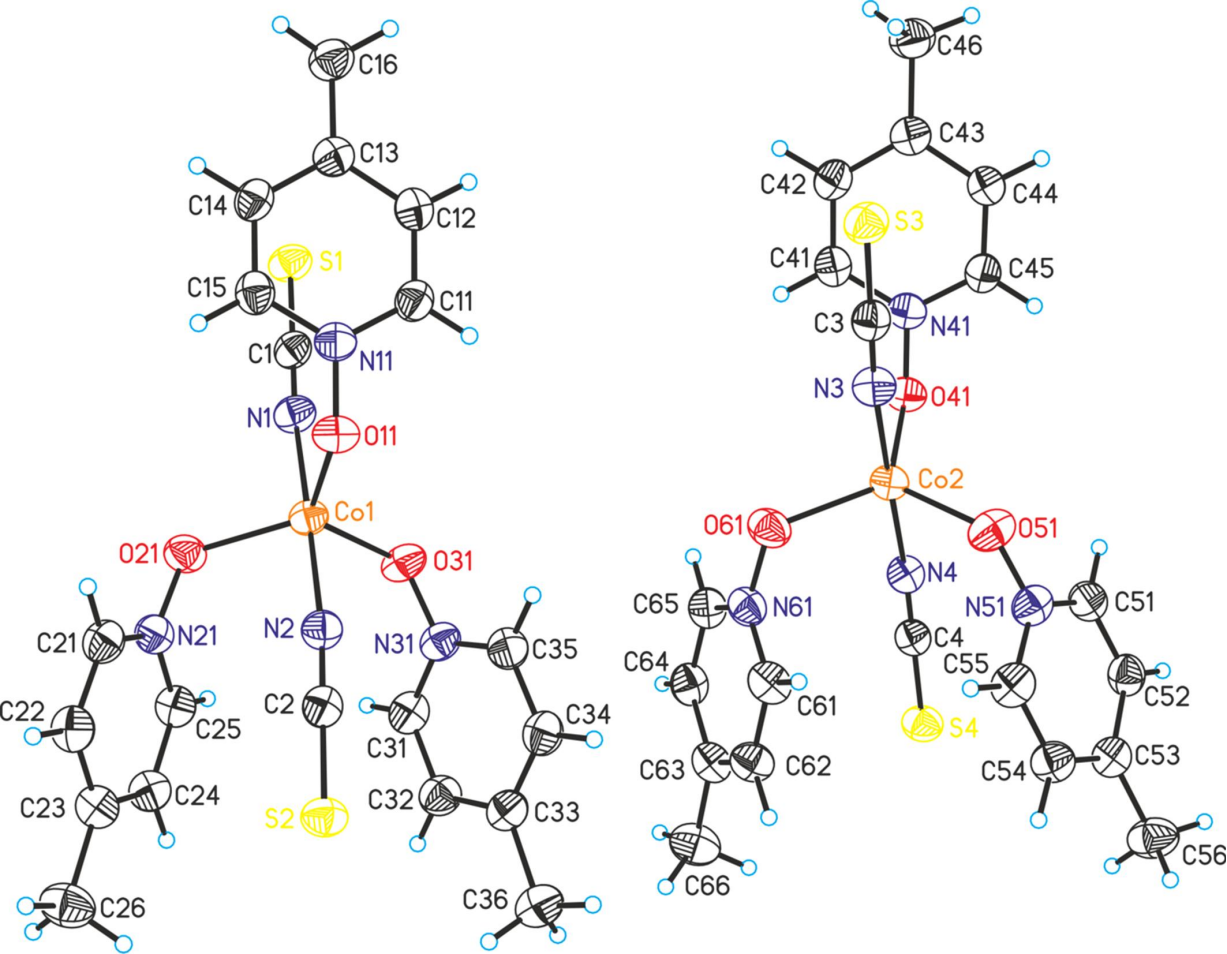
Crystal structure of the two crystallographically independent complexes mol­ecules in compound **2**, with displacement ellipsoids drawn at the 50% probability level.

In compound **2**, all the atoms are in general positions and two crystallographically independent discrete complexes are present (Fig. 2 ▸). In each of them, the Co^II^ cations are fivefold coordinated by two terminal *N*-bonded thio­cyanate anions and three 4-methyl­pyridine *N*-oxide coligands, and the coordination polyhedra around the Co centres can be described as slightly distorted trigonal pyramids with the anionic ligands in the axial and the coligands in the equatorial positions (Fig. 2 ▸ and Table 2 ▸). Within each complex, two of the coligands point ‘up’ (roughly parallel to the axis of the Co—NCS grouping) and one points ‘down’. Bond lengths and angles are comparable in both complexes (Table 2 ▸). As mentioned above, Co(NCS)_2_ complexes with a fivefold coordination are relatively rare and, therefore, it is surprising that compound **2** can be prepared easily, which is not the case for **1** with an octa­hedral coordination (see *Synthesis and crystallization* section).

## 3. Supra­molecular features

**Figure 3:**
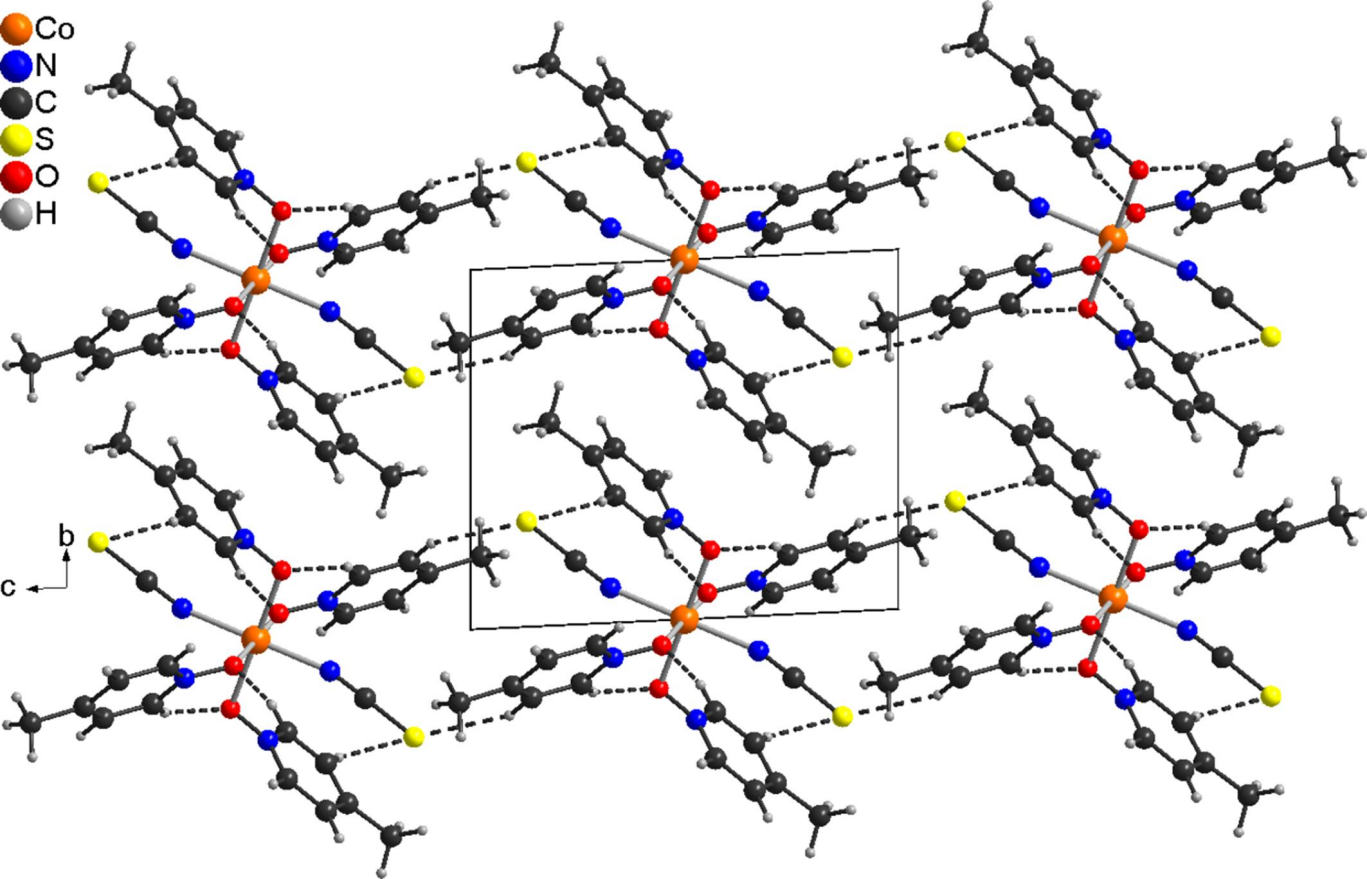
Crystal structure of compound **1**, viewed along the crystallographic *a* axis. Inter­molecular C—H⋯S and C—H⋯O contacts are shown as dashed lines.

**Figure 4:**
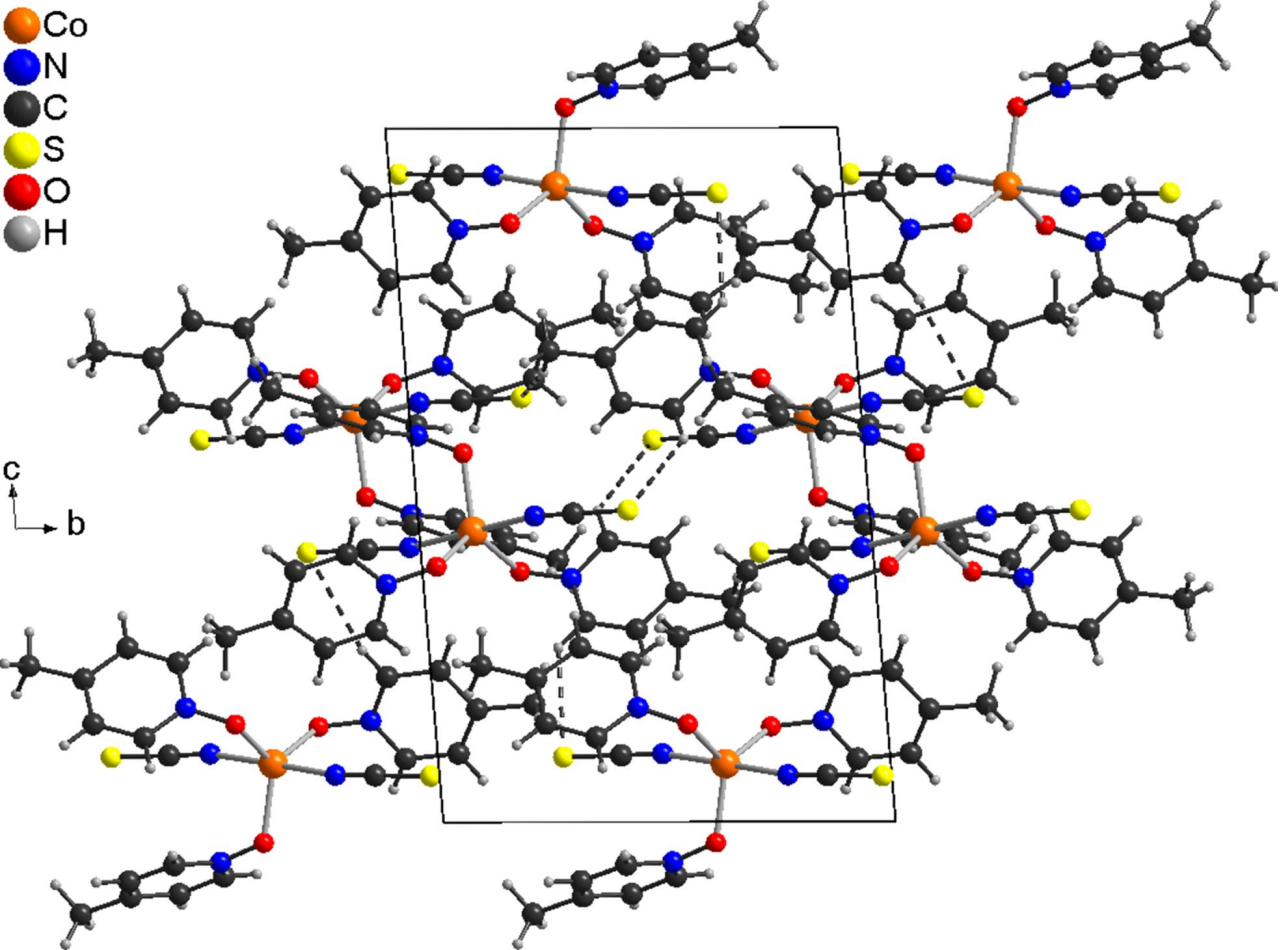
Crystal structure of compound **2**, viewed along the crystallographic *a* axis. Inter­molecular C—H⋯S contacts are shown as dashed lines.

In the extended structure of **1**, the discrete complex mol­ecules are arranged into columns that proceed along the *a*-axis direction (Fig. 3 ▸). Between the complexes, weak inter­molecular C—H⋯O and C—H⋯S inter­actions are observed (Table 3 ▸). In compound **2**, numerous C—H⋯O, C—H⋯N and C—H⋯S inter­actions are observed, but in most of them, the *X*⋯H distances are long and the angles vary far from linearity, indicating that these are very weak inter­actions (Table 4 ▸). Some C—H⋯S contacts seems to be stronger, and if they are considered, the discrete complexes are linked into chains (Fig. 4 ▸).

## 4. Database survey

A CSD search for cobalt thio­cyanate compounds with pyridine *N*-oxide derivatives revealed that only a few structures have been reported. These include discrete complexes with the composition Co(NCS)_2_(pyridine *N*-oxide)_2_(H_2_O)_2_ (FONBIU; Shi *et al.*, 2005*b* ▸) and Co(NCS)_2_(3-hy­droxy­pyridine *N*-oxide)_2_(H_2_O)_2_ (IDOYEG; Shi *et al.*, 2006*e* ▸), in which the Co cations are octa­hedrally coordinated by two thio­cyanate anions, two water mol­ecules and two terminal 3-hy­droxy­pyridine *N*-oxide ligands. Discrete dinuclear complexes are observed in Co(NCS)_2_(2-pyridine­carboxaldehyde-1-oxido 2′-pyridinylhydrazone), in which the thio­cyanate anions are only terminally *N*-bonded and two Co^II^ cations are linked by two μ-1,1-bridging O atoms of the coligands (VAZDAB; Craig *et al.*, 1989 ▸).

In Co(NCS)_2_[*N*,*N*′-ethane-1,2-diylbis(pyridine-4-carboxamide) 1,1-dioxide](H_2_O)_2_ dihydrate (FATJAN; Cao *et al.*, 2012 ▸) and in Co(NCS)_2_[*N*,*N*′-hexane-1,6-diylbis(pyridine-4-carboxamide) 1,1′-dioxide](H_2_O)_2_ (FATJER; Cao *et al.*, 2012 ▸), the Co cations are also octa­hedrally coordinated but linked into chains by the pyridine *N*-oxide coligands.

In Co(NCS)_2_(4-nitro­pyridine *N*-oxide)_2_, the Co^II^ cations are octa­hedrally coordinated by four bridging thio­cyanate anions and two terminal *O*-bonded 4-nitro­pyridine *N*-oxide coligands and linked by pairs of thio­cyanate anions into chains (TILHIG; Shi *et al.*, 2007 ▸). Chains are also observed in Co(NCS)_2_(4-methyl­pyridine *N*-oxide)(methanol) (REKBUF; Shi *et al.*, 2006*a* ▸).

A layered structure is observed in Co(NCS)_2_(4-meth­oxy­pyridine)_2_ (TERRAK; Zhang *et al.*, 2006*a* ▸). In this structure, the Co^II^ cations are octa­hedrally coordinated by four bridging anionic ligands and two coligands. As in Co(NCS)_2_(2-methyl­pyridine *N*-oxide)(methanol), the cations are con­nected into chains by alternating pairs of thio­cyanate anions and 2-methyl­pyridine *N*-oxide coligands, and the chains are are further linked into layers by additional pairs of thio­cyanate anions. A further layered structure is found in Co(NCS)_2_(4-methyl­pyridine *N*-oxide), in which the Co^II^ cations are octa­hedrally coordinated by two *N*- and two *S*-bonding thio­cyanate anions, and two bridging 4-methyl­pyridine *N*-oxide coligand (MEQKOJ; Zhang *et al.*, 2006*b* ▸). The cations are connected by pairs of bridging thio­cyanate anions into corrugated chains, that are further linked into layers by bridging 4-methyl­pyridine *N*-oxide coligands.

In Co(NCS)_2_(1,3-bis­(4-pyrid­yl)propane *N*,*N*′-dioxide)(H_2_O)_2_, each two Co^II^ cations are further linked by μ-1,1-bridging atoms of the *N*-oxide ligands into dinuclear units that are futher connected into layers by the 1,3-bis­(4-pyrid­yl)propane *N*,*N*′-dioxide coligands (UMAVAF; Zhang *et al.*, 2003 ▸). Layers are also observed in Co(NCS)_2_(1,3-bis­(4-pyrid­yl)propane *N*,*N*′-dioxide)_2_, in which the Co^II^ cations are also linked by the *N*-oxide coligands (UMAVUZ; Zhang *et al.*, 2003 ▸).

Finally we note that some compounds with 4-methyl­pyridine *N*-oxide and other transition-metal cations are reported in the CSD. These include discrete octa­hedral complexes with the composition *M*(NCS)_2_(4-methyl­pyridine *N*-oxide)_2_(H_2_O)_2_, with *M* = Mn (KESSEJ; Mautner *et al.*, 2018*a* ▸) and Ni (GAMDOO; Shi *et al.*, 2005*a* ▸). These also include Ni(NCS)_2_(4-methyl­pyridine *N*-oxide) [PEDSUN (Shi *et al.*, 2006*c* ▸) and PETSUN01 (Marsh, 2009 ▸)]. There is also one Cu compound with the composition Cu(NCS)2(4-methyl­pyridine) (TEBTAW; Shi *et al.*, 2006*d* ▸) and one Cd compound with the composition Cd(NCS)_2_(4-methyl­pyridine) (TEQKAC; Shi *et al.*, 2006*b* ▸), in which the cations are linked into chains.

## 5. Additional investigations

Based on the single-crystal data, a powder pattern was calculated and compared with the experimental pattern, which revealed that compound **2** was nearly obtained as a pure phase (Fig. S1 in the supporting information). There are a few additional reflections of very low intensity that cannot be assigned to a known phase.

The thermal behaviour of compound **2** was investigated by thermogravimetry and differential thermoanalysis (TG–DTA) measurements. Upon heating at a rate of 8 K min^−1^, one mass loss is observed, accompanied by an exothermic event in the DTA curve (Fig. S2). The experimental mass loss of 68.1% is in reasonable agreement with that calculated for the removal of all three 4-methyl­pyridine *N*-oxide coligands of 65.2%. The exothermic signal, however, indicates that the coligand decompose as already observed for compounds with other pyridine *N*-oxide derivatives (Näther & Jess, 2023 ▸). There is one endothermic signal at 438 K, where the sample mass does not change, which might originate from a melting of the complex before decomposition is observed.

## 6. Synthesis and crystallization

Co(NCS)_2_ (99%) was purchased from Sigma–Aldrich and 4-methyl­pyridine *N*-oxide (98%) from Fisher Chemical. Single crystals of compound **2** were obtained by the reaction of Co(SCN)_2_ (0.500 mmol, 87.5 mg) and 4-methyl­pyridine *N*-oxide (1.500 mmol, 163.7 mg) in methanol (1 ml). Within 2 d, crystals suitable for structure analysis were obtained. If the same reaction conditions are used and the batch is stirred for 1 d, a microcrystalline powder of **2** is obtained.

For compound **1**, a few crystals were obtained accidentally in a mixture with **2**, using the same conditions as described above. It is noted that **2** is also obtained if Co(NCS)_2_ is reacted with 4-methyl­pyridine *N*-oxide in a 1:4 ratio. We also used larger ratios and other solvents, *e.g.* ethanol or *n*-butanol, but in none of these batches was compound **1** obtained as a pure phase. It seems that compound **2**, with a fivefold coordination, is more stable. Finally, it is noted that in some batches where methanol and ethanol was used as solvent, powder X-ray diffraction (PXRD) measurements prove that additional and unknown crystalline phases were obtained.

The PXRD data were collected using an XtaLAB Synergy, Dualflex, Thermogravimetry and differential thermoanalysis (TG–DTA) measurements were performed under a dynamic nitro­gen atmosphere in Al_2_O_3_ crucibles using an STA-PT 1000 thermobalance from Linseis. The instrument was calibrated using standard reference materials.

## 7. Refinement

The H atoms were positioned with idealized geometry (C—H = 0.95–0.98 Å) and were refined using a riding model, with *U*_iso_(H) = 1.2*U*_eq_(C) or 1.5*U*_eq_(methyl C).

The crystal of **2** chosen for data collection was found to be twinned. Both components were indexed separately (Fig. S3) and afterwards a twin-refinement with data in HKLF-5 format using the twin matrix −0.9998 0.0004 −0.0001/−0.0006 −1.0001 −0.0006/0.2122 0.2564 1.0002 was performed. Therefore, no inter­nal *R* value is reported. The ratio between domains refined to 0.8273 (7):0.1727 (7). Crystal data, data collection and structure refinement details are summarized in Table 5 ▸.

## Supplementary Material

Crystal structure: contains datablock(s) global, 2, 1. DOI: 10.1107/S2056989024000471/hb8092sup1.cif

Structure factors: contains datablock(s) 1. DOI: 10.1107/S2056989024000471/hb80921sup2.hkl

Structure factors: contains datablock(s) 2. DOI: 10.1107/S2056989024000471/hb80922sup3.hkl

Additional figures. DOI: 10.1107/S2056989024000471/hb8092sup4.pdf

CCDC references: 2325197, 2325196

Additional supporting information:  crystallographic information; 3D view; checkCIF report

## Figures and Tables

**Table 1:** Selected geometric parameters (Å, °) for (**1**)

Co1—N1	2.0910 (14)	Co1—O21	2.1266 (11)
Co1—O11	2.1005 (12)		
			
N1^i^—Co1—O11^i^	92.39 (5)	O11^i^—Co1—O21^i^	87.15 (5)
N1—Co1—O11^i^	87.61 (5)	O11^i^—Co1—O21	92.85 (5)
N1—Co1—O21^i^	87.56 (5)	Co1—N1—C1	165.56 (14)
N1^i^—Co1—O21^i^	92.44 (5)		

Symmetry code: (i) 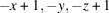 .

**Table 2:** Selected geometric parameters (Å, °) for (**2**)

Co1—N1	2.0767 (16)	Co2—N3	2.0804 (16)
Co1—N2	2.0895 (15)	Co2—N4	2.0844 (16)
Co1—O11	1.9949 (13)	Co2—O41	1.9999 (13)
Co1—O21	1.9989 (14)	Co2—O51	1.9880 (14)
Co1—O31	2.0045 (14)	Co2—O61	1.9941 (14)
			
N1—Co1—N2	179.11 (7)	O41—Co2—N3	93.31 (6)
O11—Co1—N1	93.86 (6)	O41—Co2—N4	88.08 (6)
O11—Co1—N2	86.87 (6)	O51—Co2—N3	86.15 (6)
O11—Co1—O21	122.93 (6)	O51—Co2—N4	92.14 (6)
O11—Co1—O31	121.98 (6)	O51—Co2—O41	122.04 (6)
O21—Co1—N1	87.05 (6)	O51—Co2—O61	115.95 (6)
O21—Co1—N2	92.13 (6)	O61—Co2—N3	87.50 (6)
O21—Co1—O31	115.07 (6)	O61—Co2—N4	92.75 (6)
O31—Co1—N1	87.43 (6)	O61—Co2—O41	121.94 (6)
O31—Co1—N2	92.62 (6)	Co2—N3—Co3	168.81 (15)
Co1—N1—Co	167.85 (16)		

**Table 3:** Hydrogen-bond geometry (Å, °) for (**1**)

*D*—H⋯*A*	*D*—H	H⋯*A*	*D*⋯*A*	*D*—H⋯*A*
C14—H14⋯S1^ii^	0.95	2.83	3.7271 (17)	157
C15—H15⋯O21^iii^	0.95	2.40	3.295 (2)	157
C21—H21⋯O21^iii^	0.95	2.62	3.520 (2)	158
C24—H24⋯S1^iv^	0.95	2.87	3.7591 (18)	156
C25—H25⋯O11^i^	0.95	2.25	3.090 (2)	147

Symmetry codes: (i) 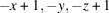 ; (ii) 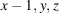 ; (iii) 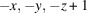 ; (iv) 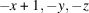 .

**Table 4:** Hydrogen-bond geometry (Å, °) for (**2**)

*D*—H⋯*A*	*D*—H	H⋯*A*	*D*⋯*A*	*D*—H⋯*A*
C11—H11⋯S4^i^	0.95	2.88	3.8088 (19)	166
C21—H21⋯S2^ii^	0.95	2.87	3.739 (2)	153
C24—H24⋯S1^iii^	0.95	2.90	3.841 (2)	169
C25—H25⋯O31^iii^	0.95	2.63	3.259 (2)	124
C31—H31⋯O21^iii^	0.95	2.44	3.277 (2)	146
C34—H34⋯S3	0.95	2.99	3.754 (2)	138
C41—H41⋯S3^iv^	0.95	2.97	3.6966 (19)	134
C45—H45⋯S2	0.95	2.89	3.6082 (19)	133
C55—H55⋯O61^v^	0.95	2.46	3.184 (2)	133
C61—H61⋯O51^v^	0.95	2.48	3.233 (2)	136
C62—H62⋯S3^v^	0.95	2.98	3.900 (2)	162
C64—H64⋯N4^vi^	0.95	2.64	3.484 (2)	148
C65—H65⋯S4^vi^	0.95	3.00	3.870 (2)	154

Symmetry codes: (i) 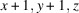 ; (ii) 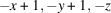 ; (iii) 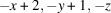 ; (iv) 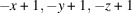 ; (v) 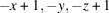 ; (vi) 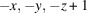 .

**Table 5:** Experimental details For both structures: triclinic, *P* 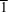 . Experiments were carried out at 100 K with Cu *K*α radiation using a Rigaku XtaLAB Synergy Dualflex diffractometer with a HyPix detector. Absorption was corrected for by multi-scan methods, (*CrysAlis PRO*; Rigaku OD, 2021 ▸). H-atom parameters were constrained.

	**1**	**2**
Crystal data		
Chemical formula	[Co(NCS)_2_(C_6_H_7_NO)_4_]	[Co(NCS)_2_(C_6_H_7_NO)_3_]
*M*_r_	611.59	502.47
*a*, *b*, *c* (Å)	7.0709 (3), 9.6651 (5), 11.0401 (4)	11.70330 (8), 12.55284 (9), 17.5256 (2)
α, β, γ (°)	90.609 (3), 96.346 (3), 108.090 (4)	93.5044 (8), 91.8625 (8), 115.0705 (7)
*V* (Å^3^)	712.00 (6)	2322.89 (4)
*Z*	1	4
μ (mm^−1^)	6.45	7.74
Crystal size (mm)	0.12 × 0.08 × 0.04	0.2 × 0.18 × 0.1

Data collection		
*T*_min_, *T*_max_	0.859, 1.000	0.024, 0.116
No. of measured, independent and observed [*I* > 2σ(*I*)] reflections	7433, 2950, 2935	11225, 11225, 10980
*R*_int_	0.017	See *Refinement* section
(sin θ/λ)_max_ (Å^−1^)	0.639	0.639

Refinement		
*R*[*F* ^2^ > 2σ(*F* ^2^)], *wR*(*F* ^2^), *S*	0.029, 0.081, 1.11	0.029, 0.081, 1.07
No. of reflections	2950	11225
No. of parameters	181	566
Δρ_max_, Δρ_min_ (e Å^−3^)	0.34, −0.48	0.33, −0.25

Computer programs: *CrysAlis PRO* (Rigaku OD, 2021 ▸), *SHELXT2014* (Sheldrick, 2015*b* ▸), *SHELXL2016* (Sheldrick, 2015*a* ▸), *DIAMOND* (Brandenburg & Putz, 1999 ▸) and *publCIF* (Westrip, 2010 ▸).

## supplementary crystallographic information

### Tetrakis(4-methylpyridine N-oxide-κO)bis(thiocyanato-κN)cobalt(II) (1) Crystal data

**Table:**

[Co(NCS)_2_(C_6_H_7_NO)_4_]	*Z* = 1
*M**_r_* = 611.59	*F*(000) = 317
Triclinic, *P*1	*D*_x_ = 1.426 Mg m^−^^3^
*a* = 7.0709 (3) Å	Cu *K*α radiation, λ = 1.54184 Å
*b* = 9.6651 (5) Å	Cell parameters from 5312 reflections
*c* = 11.0401 (4) Å	θ = 4.0–78.4°
α = 90.609 (3)°	µ = 6.45 mm^−^^1^
β = 96.346 (3)°	*T* = 100 K
γ = 108.090 (4)°	Block, violet
*V* = 712.00 (6) Å^3^	0.12 × 0.08 × 0.04 mm

### Tetrakis(4-methylpyridine N-oxide-κO)bis(thiocyanato-κN)cobalt(II) (1) Data collection

**Table:**

Rigaku XtaLAB Synergy Dualflex diffractometer with a HyPix detector	2950 independent reflections
Radiation source: micro-focus sealed X-ray tube, PhotonJet (Cu) X-ray Source	2935 reflections with *I* > 2σ(*I*)
Mirror monochromator	*R*_int_ = 0.017
Detector resolution: 10.0000 pixels mm^-1^	θ_max_ = 79.9°, θ_min_ = 4.0°
ω scans	*h* = −8→6
Absorption correction: multi-scan (CrysAlis PRO; Rigaku OD, 2021)	*k* = −12→12
*T*_min_ = 0.859, *T*_max_ = 1.000	*l* = −13→14
7433 measured reflections	

### Tetrakis(4-methylpyridine N-oxide-κO)bis(thiocyanato-κN)cobalt(II) (1) Refinement

**Table:**

Refinement on *F*^2^	Hydrogen site location: inferred from neighbouring sites
Least-squares matrix: full	H-atom parameters constrained
*R*[*F*^2^ > 2σ(*F*^2^)] = 0.029	*w* = 1/[σ^2^(*F*_o_^2^) + (0.0431*P*)^2^ + 0.3098*P*] where *P* = (*F*_o_^2^ + 2*F*_c_^2^)/3
*wR*(*F*^2^) = 0.081	(Δ/σ)_max_ < 0.001
*S* = 1.11	Δρ_max_ = 0.34 e Å^−^^3^
2950 reflections	Δρ_min_ = −0.47 e Å^−^^3^
181 parameters	Extinction correction: SHELXL2016 (Sheldrick, 2015a), Fc^*^=kFc[1+0.001xFc^2^λ^3^/sin(2θ)]^-1/4^
0 restraints	Extinction coefficient: 0.0030 (6)
Primary atom site location: dual	

### Tetrakis(4-methylpyridine N-oxide-κO)bis(thiocyanato-κN)cobalt(II) (1) Special details

**Table:**

Geometry. All esds (except the esd in the dihedral angle between two l.s. planes) are estimated using the full covariance matrix. The cell esds are taken into account individually in the estimation of esds in distances, angles and torsion angles; correlations between esds in cell parameters are only used when they are defined by crystal symmetry. An approximate (isotropic) treatment of cell esds is used for estimating esds involving l.s. planes.

### Tetrakis(4-methylpyridine N-oxide-κO)bis(thiocyanato-κN)cobalt(II) (1) Fractional atomic coordinates and isotropic or equivalent isotropic displacement parameters (Å^2^)

**Table:**

	*x*	*y*	*z*	*U*_iso_*/*U*_eq_	
Co1	0.500000	0.000000	0.500000	0.01633 (12)	
N1	0.4838 (2)	−0.09719 (16)	0.32743 (12)	0.0207 (3)	
C1	0.4728 (2)	−0.17698 (19)	0.24550 (14)	0.0205 (3)	
S1	0.45856 (6)	−0.29171 (5)	0.13159 (4)	0.02849 (13)	
O11	0.29908 (17)	−0.18891 (13)	0.55992 (10)	0.0207 (2)	
N11	0.1767 (2)	−0.28091 (15)	0.47249 (12)	0.0177 (3)	
C11	0.2312 (3)	−0.39113 (19)	0.42796 (16)	0.0232 (3)	
H11	0.354482	−0.404151	0.460193	0.028*	
C12	0.1101 (3)	−0.48501 (19)	0.33621 (16)	0.0252 (4)	
H12	0.149798	−0.562764	0.305869	0.030*	
C13	−0.0708 (3)	−0.46695 (19)	0.28735 (15)	0.0231 (3)	
C14	−0.1231 (2)	−0.35312 (19)	0.33727 (15)	0.0216 (3)	
H14	−0.246404	−0.338563	0.307292	0.026*	
C15	0.0012 (2)	−0.26130 (18)	0.42952 (15)	0.0193 (3)	
H15	−0.036625	−0.184181	0.462878	0.023*	
C16	−0.2018 (3)	−0.5642 (2)	0.18404 (18)	0.0328 (4)	
H16A	−0.130107	−0.552506	0.111847	0.049*	
H16B	−0.324611	−0.537857	0.165466	0.049*	
H16C	−0.236267	−0.665831	0.207245	0.049*	
O21	0.24737 (17)	0.07014 (13)	0.44699 (10)	0.0207 (2)	
N21	0.1883 (2)	0.09747 (15)	0.33301 (12)	0.0186 (3)	
C21	−0.0100 (2)	0.06152 (18)	0.29449 (15)	0.0202 (3)	
H21	−0.104965	0.014060	0.347311	0.024*	
C22	−0.0750 (2)	0.09351 (19)	0.17864 (15)	0.0217 (3)	
H22	−0.214644	0.067556	0.152642	0.026*	
C23	0.0608 (3)	0.16314 (19)	0.09945 (15)	0.0226 (3)	
C24	0.2635 (3)	0.1974 (2)	0.14277 (16)	0.0258 (4)	
H24	0.360977	0.244184	0.091201	0.031*	
C25	0.3255 (2)	0.1652 (2)	0.25860 (16)	0.0236 (4)	
H25	0.464495	0.190292	0.286400	0.028*	
C26	−0.0054 (3)	0.2016 (2)	−0.02611 (16)	0.0284 (4)	
H26A	−0.131570	0.128174	−0.058861	0.043*	
H26B	0.097434	0.204362	−0.079528	0.043*	
H26C	−0.025290	0.297392	−0.021875	0.043*	

### Tetrakis(4-methylpyridine N-oxide-κO)bis(thiocyanato-κN)cobalt(II) (1) Atomic displacement parameters (Å^2^)

**Table:**

	*U*^11^	*U*^22^	*U*^33^	*U*^12^	*U*^13^	*U*^23^
Co1	0.01222 (18)	0.0232 (2)	0.01451 (18)	0.00671 (14)	0.00198 (12)	0.00032 (13)
N1	0.0185 (7)	0.0275 (7)	0.0160 (6)	0.0071 (5)	0.0021 (5)	−0.0012 (5)
C1	0.0131 (7)	0.0322 (9)	0.0179 (7)	0.0095 (6)	0.0013 (6)	0.0045 (6)
S1	0.0247 (2)	0.0448 (3)	0.0193 (2)	0.01733 (19)	−0.00083 (15)	−0.00852 (17)
O11	0.0159 (5)	0.0261 (6)	0.0171 (5)	0.0032 (4)	−0.0003 (4)	0.0014 (4)
N11	0.0152 (6)	0.0216 (7)	0.0152 (6)	0.0042 (5)	0.0022 (5)	0.0016 (5)
C11	0.0197 (8)	0.0276 (9)	0.0261 (8)	0.0118 (7)	0.0054 (6)	0.0047 (7)
C12	0.0284 (9)	0.0233 (8)	0.0269 (8)	0.0107 (7)	0.0085 (7)	0.0015 (7)
C13	0.0263 (9)	0.0223 (8)	0.0193 (8)	0.0050 (7)	0.0039 (6)	0.0026 (6)
C14	0.0190 (8)	0.0250 (8)	0.0206 (8)	0.0076 (6)	−0.0004 (6)	0.0026 (6)
C15	0.0178 (7)	0.0227 (8)	0.0187 (7)	0.0080 (6)	0.0026 (6)	0.0012 (6)
C16	0.0377 (11)	0.0256 (9)	0.0305 (9)	0.0055 (8)	−0.0017 (8)	−0.0054 (7)
O21	0.0191 (5)	0.0319 (6)	0.0146 (5)	0.0127 (5)	0.0026 (4)	0.0039 (4)
N21	0.0172 (6)	0.0250 (7)	0.0160 (6)	0.0101 (5)	0.0011 (5)	0.0017 (5)
C21	0.0145 (7)	0.0241 (8)	0.0240 (8)	0.0085 (6)	0.0040 (6)	−0.0004 (6)
C22	0.0174 (8)	0.0263 (8)	0.0236 (8)	0.0115 (6)	−0.0006 (6)	−0.0008 (6)
C23	0.0224 (8)	0.0269 (9)	0.0222 (8)	0.0133 (7)	0.0016 (6)	0.0014 (6)
C24	0.0197 (8)	0.0359 (10)	0.0229 (8)	0.0096 (7)	0.0052 (6)	0.0060 (7)
C25	0.0148 (7)	0.0334 (9)	0.0233 (8)	0.0081 (7)	0.0036 (6)	0.0063 (7)
C26	0.0269 (9)	0.0390 (10)	0.0228 (8)	0.0157 (8)	0.0008 (7)	0.0055 (7)

### Tetrakis(4-methylpyridine N-oxide-κO)bis(thiocyanato-κN)cobalt(II) (1) Geometric parameters (Å, º)

**Table:**

Co1—N1	2.0910 (14)	C15—H15	0.9500
Co1—N1^i^	2.0910 (14)	C16—H16A	0.9800
Co1—O11	2.1005 (12)	C16—H16B	0.9800
Co1—O11^i^	2.1005 (12)	C16—H16C	0.9800
Co1—O21^i^	2.1266 (11)	O21—N21	1.3369 (17)
Co1—O21	2.1266 (11)	N21—C21	1.352 (2)
N1—C1	1.163 (2)	N21—C25	1.356 (2)
C1—S1	1.6414 (18)	C21—H21	0.9500
O11—N11	1.3381 (17)	C21—C22	1.382 (2)
N11—C11	1.346 (2)	C22—H22	0.9500
N11—C15	1.348 (2)	C22—C23	1.391 (2)
C11—H11	0.9500	C23—C24	1.393 (2)
C11—C12	1.374 (3)	C23—C26	1.501 (2)
C12—H12	0.9500	C24—H24	0.9500
C12—C13	1.394 (3)	C24—C25	1.376 (2)
C13—C14	1.392 (3)	C25—H25	0.9500
C13—C16	1.498 (2)	C26—H26A	0.9800
C14—H14	0.9500	C26—H26B	0.9800
C14—C15	1.377 (2)	C26—H26C	0.9800
			
N1—Co1—N1^i^	180.00 (8)	N11—C15—H15	120.0
N1^i^—Co1—O11^i^	92.39 (5)	C14—C15—H15	120.0
N1—Co1—O11^i^	87.61 (5)	C13—C16—H16A	109.5
N1^i^—Co1—O11	87.61 (5)	C13—C16—H16B	109.5
N1—Co1—O11	92.39 (5)	C13—C16—H16C	109.5
N1—Co1—O21^i^	87.56 (5)	H16A—C16—H16B	109.5
N1^i^—Co1—O21^i^	92.44 (5)	H16A—C16—H16C	109.5
N1^i^—Co1—O21	87.56 (5)	H16B—C16—H16C	109.5
N1—Co1—O21	92.44 (5)	N21—O21—Co1	125.03 (9)
O11—Co1—O11^i^	180.00 (5)	O21—N21—C21	119.16 (13)
O11^i^—Co1—O21^i^	87.15 (5)	O21—N21—C25	120.30 (13)
O11—Co1—O21	87.15 (5)	C21—N21—C25	120.50 (14)
O11^i^—Co1—O21	92.85 (5)	N21—C21—H21	119.9
O11—Co1—O21^i^	92.85 (5)	N21—C21—C22	120.29 (15)
O21^i^—Co1—O21	180.0	C22—C21—H21	119.9
Co1—N1—C1	165.56 (14)	C21—C22—H22	119.5
N1—C1—S1	178.94 (16)	C21—C22—C23	121.07 (15)
N11—O11—Co1	115.97 (9)	C23—C22—H22	119.5
O11—N11—C11	119.50 (14)	C22—C23—C24	116.66 (15)
O11—N11—C15	119.60 (14)	C22—C23—C26	122.24 (16)
C11—N11—C15	120.90 (14)	C24—C23—C26	121.09 (16)
N11—C11—H11	119.7	C23—C24—H24	119.3
N11—C11—C12	120.53 (16)	C25—C24—C23	121.49 (16)
C12—C11—H11	119.7	C25—C24—H24	119.3
C11—C12—H12	119.8	N21—C25—C24	119.99 (15)
C11—C12—C13	120.48 (16)	N21—C25—H25	120.0
C13—C12—H12	119.8	C24—C25—H25	120.0
C12—C13—C16	121.54 (17)	C23—C26—H26A	109.5
C14—C13—C12	117.17 (16)	C23—C26—H26B	109.5
C14—C13—C16	121.28 (16)	C23—C26—H26C	109.5
C13—C14—H14	119.5	H26A—C26—H26B	109.5
C15—C14—C13	120.93 (16)	H26A—C26—H26C	109.5
C15—C14—H14	119.5	H26B—C26—H26C	109.5
N11—C15—C14	119.97 (15)		

Symmetry code: (i) −*x*+1, −*y*, −*z*+1.

### Tetrakis(4-methylpyridine N-oxide-κO)bis(thiocyanato-κN)cobalt(II) (1) Hydrogen-bond geometry (Å, º)

**Table:**

*D*—H···*A*	*D*—H	H···*A*	*D*···*A*	*D*—H···*A*
C14—H14···S1^ii^	0.95	2.83	3.7271 (17)	157
C15—H15···O21^iii^	0.95	2.40	3.295 (2)	157
C21—H21···O21^iii^	0.95	2.62	3.520 (2)	158
C24—H24···S1^iv^	0.95	2.87	3.7591 (18)	156
C25—H25···O11^i^	0.95	2.25	3.090 (2)	147

Symmetry codes: (i) −*x*+1, −*y*, −*z*+1; (ii) *x*−1, *y*, *z*; (iii) −*x*, −*y*, −*z*+1; (iv) −*x*+1, −*y*, −*z*.

### Tris(4-methylpyridine N-oxide-κO)bis(thiocyanato-κN)cobalt(II) (2) Crystal data

**Table:**

[Co(NCS)_2_(C_6_H_7_NO)_3_]	*Z* = 4
*M**_r_* = 502.47	*F*(000) = 1036
Triclinic, *P*1	*D*_x_ = 1.437 Mg m^−^^3^
*a* = 11.70330 (8) Å	Cu *K*α radiation, λ = 1.54184 Å
*b* = 12.55284 (9) Å	Cell parameters from 22375 reflections
*c* = 17.5256 (2) Å	θ = 3.9–79.4°
α = 93.5044 (8)°	µ = 7.74 mm^−^^1^
β = 91.8625 (8)°	*T* = 100 K
γ = 115.0705 (7)°	Block, violet
*V* = 2322.89 (4) Å^3^	0.2 × 0.18 × 0.1 mm

### Tris(4-methylpyridine N-oxide-κO)bis(thiocyanato-κN)cobalt(II) (2) Data collection

**Table:**

Rigaku XtaLAB Synergy Dualflex diffractometer with a HyPix detector	11225 measured reflections
Radiation source: micro-focus sealed X-ray tube, PhotonJet (Cu) X-ray Source	11225 independent reflections
Mirror monochromator	10980 reflections with *I* > 2σ(*I*)
Detector resolution: 10.0000 pixels mm^-1^	θ_max_ = 80.3°, θ_min_ = 2.5°
ω scans	*h* = −14→14
Absorption correction: multi-scan (CrysAlis PRO; Rigaku OD, 2021)	*k* = −15→15
*T*_min_ = 0.024, *T*_max_ = 0.116	*l* = −22→22

### Tris(4-methylpyridine N-oxide-κO)bis(thiocyanato-κN)cobalt(II) (2) Refinement

**Table:**

Refinement on *F*^2^	Primary atom site location: dual
Least-squares matrix: full	Hydrogen site location: inferred from neighbouring sites
*R*[*F*^2^ > 2σ(*F*^2^)] = 0.029	H-atom parameters constrained
*wR*(*F*^2^) = 0.081	*w* = 1/[σ^2^(*F*_o_^2^) + (0.045*P*)^2^ + 0.8076*P*] where *P* = (*F*_o_^2^ + 2*F*_c_^2^)/3
*S* = 1.07	(Δ/σ)_max_ = 0.001
11225 reflections	Δρ_max_ = 0.33 e Å^−^^3^
566 parameters	Δρ_min_ = −0.25 e Å^−^^3^
0 restraints	

### Tris(4-methylpyridine N-oxide-κO)bis(thiocyanato-κN)cobalt(II) (2) Special details

**Table:**

Geometry. All esds (except the esd in the dihedral angle between two l.s. planes) are estimated using the full covariance matrix. The cell esds are taken into account individually in the estimation of esds in distances, angles and torsion angles; correlations between esds in cell parameters are only used when they are defined by crystal symmetry. An approximate (isotropic) treatment of cell esds is used for estimating esds involving l.s. planes.
Refinement. Refined as a 2-component twin.

### Tris(4-methylpyridine N-oxide-κO)bis(thiocyanato-κN)cobalt(II) (2) Fractional atomic coordinates and isotropic or equivalent isotropic displacement parameters (Å^2^)

**Table:**

	*x*	*y*	*z*	*U*_iso_*/*U*_eq_	
Co1	0.87727 (3)	0.63471 (2)	0.08404 (2)	0.03107 (7)	
N1	1.05248 (15)	0.77002 (14)	0.06799 (10)	0.0364 (3)	
C1	1.14327 (17)	0.85769 (17)	0.06745 (11)	0.0340 (4)	
S1	1.27208 (4)	0.98014 (4)	0.06637 (3)	0.04113 (11)	
N2	0.70108 (14)	0.49727 (14)	0.09855 (9)	0.0336 (3)	
C2	0.61353 (17)	0.40560 (16)	0.09628 (10)	0.0332 (4)	
S2	0.49072 (4)	0.27783 (4)	0.09380 (3)	0.03765 (10)	
O11	0.81378 (12)	0.74195 (11)	0.13747 (8)	0.0370 (3)	
N11	0.89135 (14)	0.85821 (13)	0.14616 (9)	0.0333 (3)	
C11	0.97058 (18)	0.90240 (17)	0.20938 (11)	0.0359 (4)	
H11	0.970160	0.851990	0.247757	0.043*	
C12	1.05192 (18)	1.02021 (17)	0.21838 (11)	0.0367 (4)	
H12	1.107390	1.050856	0.263117	0.044*	
C13	1.05383 (18)	1.09533 (17)	0.16239 (11)	0.0346 (4)	
C14	0.96919 (18)	1.04603 (17)	0.09893 (11)	0.0350 (4)	
H14	0.966689	1.094846	0.060173	0.042*	
C15	0.88907 (18)	0.92801 (17)	0.09118 (11)	0.0354 (4)	
H15	0.832126	0.895551	0.047195	0.042*	
C16	1.1468 (2)	1.22268 (18)	0.16920 (13)	0.0432 (4)	
H16A	1.160465	1.253518	0.223087	0.065*	
H16B	1.113463	1.267842	0.139177	0.065*	
H16C	1.227112	1.229938	0.149776	0.065*	
O21	0.85790 (12)	0.59878 (11)	−0.02960 (8)	0.0387 (3)	
N21	0.75725 (15)	0.49966 (14)	−0.05742 (9)	0.0345 (3)	
C21	0.64766 (19)	0.50453 (18)	−0.07788 (11)	0.0380 (4)	
H21	0.642351	0.578101	−0.074644	0.046*	
C22	0.54344 (19)	0.40278 (18)	−0.10347 (12)	0.0393 (4)	
H22	0.466736	0.406627	−0.118740	0.047*	
C23	0.54960 (18)	0.29452 (18)	−0.10714 (11)	0.0380 (4)	
C24	0.66481 (18)	0.29383 (17)	−0.08511 (12)	0.0373 (4)	
H24	0.672089	0.221193	−0.086544	0.045*	
C25	0.76740 (18)	0.39666 (17)	−0.06148 (11)	0.0365 (4)	
H25	0.846042	0.395408	−0.047894	0.044*	
C26	0.4372 (2)	0.1817 (2)	−0.13225 (15)	0.0506 (5)	
H26A	0.414610	0.130513	−0.089887	0.076*	
H26B	0.457444	0.141697	−0.175943	0.076*	
H26C	0.365747	0.198853	−0.147350	0.076*	
O31	0.96976 (12)	0.55822 (12)	0.14100 (9)	0.0401 (3)	
N31	0.89880 (15)	0.45128 (14)	0.16484 (10)	0.0355 (3)	
C31	0.88804 (18)	0.35399 (18)	0.12180 (12)	0.0383 (4)	
H31	0.935574	0.361629	0.077893	0.046*	
C32	0.8084 (2)	0.24428 (18)	0.14168 (12)	0.0404 (4)	
H32	0.800278	0.176188	0.110891	0.048*	
C33	0.73886 (19)	0.23121 (17)	0.20659 (12)	0.0387 (4)	
C34	0.75831 (19)	0.33383 (18)	0.25114 (12)	0.0393 (4)	
H34	0.715986	0.328356	0.297091	0.047*	
C35	0.83775 (19)	0.44285 (17)	0.22969 (11)	0.0376 (4)	
H35	0.849641	0.512152	0.260476	0.045*	
C36	0.6449 (2)	0.1123 (2)	0.22542 (14)	0.0521 (5)	
H36A	0.576176	0.079770	0.185175	0.078*	
H36B	0.610313	0.119739	0.274605	0.078*	
H36C	0.686615	0.059380	0.228816	0.078*	
Co2	0.35615 (3)	0.11924 (2)	0.41874 (2)	0.03115 (7)	
N3	0.53440 (15)	0.25825 (14)	0.44106 (10)	0.0361 (3)	
C3	0.62725 (17)	0.34432 (16)	0.44440 (10)	0.0333 (4)	
S3	0.75865 (4)	0.46580 (4)	0.44927 (3)	0.03878 (10)	
N4	0.18025 (14)	−0.02361 (14)	0.39579 (9)	0.0343 (3)	
C4	0.09849 (17)	−0.11823 (16)	0.39280 (10)	0.0330 (4)	
S4	−0.01714 (5)	−0.25075 (4)	0.38844 (3)	0.04076 (11)	
O41	0.28977 (12)	0.21590 (11)	0.36094 (8)	0.0358 (3)	
N41	0.37181 (14)	0.32772 (14)	0.35101 (9)	0.0330 (3)	
C41	0.38679 (18)	0.41538 (17)	0.40442 (11)	0.0350 (4)	
H41	0.338588	0.398404	0.448341	0.042*	
C42	0.47149 (18)	0.52902 (17)	0.39546 (11)	0.0362 (4)	
H42	0.482020	0.590067	0.433658	0.043*	
C43	0.54250 (18)	0.55631 (17)	0.33101 (11)	0.0353 (4)	
C44	0.52331 (18)	0.46289 (17)	0.27734 (11)	0.0361 (4)	
H44	0.569378	0.477614	0.232525	0.043*	
C45	0.43865 (18)	0.34961 (17)	0.28823 (11)	0.0351 (4)	
H45	0.427369	0.286708	0.251345	0.042*	
C46	0.6381 (2)	0.67946 (18)	0.32134 (14)	0.0467 (5)	
H46A	0.604421	0.735722	0.338290	0.070*	
H46B	0.656353	0.687183	0.267249	0.070*	
H46C	0.716030	0.695957	0.352149	0.070*	
O51	0.44472 (13)	0.03239 (12)	0.36803 (9)	0.0428 (3)	
N51	0.37995 (15)	−0.08235 (14)	0.34244 (10)	0.0372 (3)	
C51	0.30902 (19)	−0.11292 (18)	0.27584 (11)	0.0380 (4)	
H51	0.304767	−0.053571	0.246299	0.046*	
C52	0.24278 (19)	−0.22988 (18)	0.25057 (11)	0.0382 (4)	
H52	0.192085	−0.251061	0.203803	0.046*	
C53	0.24931 (18)	−0.31784 (17)	0.29300 (11)	0.0375 (4)	
C54	0.32194 (19)	−0.28153 (18)	0.36173 (12)	0.0400 (4)	
H54	0.326632	−0.339102	0.392848	0.048*	
C55	0.38710 (19)	−0.16438 (18)	0.38572 (12)	0.0407 (4)	
H55	0.437167	−0.141148	0.432762	0.049*	
C56	0.1801 (2)	−0.44603 (19)	0.26705 (14)	0.0520 (5)	
H56A	0.101210	−0.480407	0.293084	0.078*	
H56B	0.232895	−0.486601	0.279491	0.078*	
H56C	0.160765	−0.455140	0.211550	0.078*	
O61	0.34565 (13)	0.11125 (12)	0.53174 (8)	0.0410 (3)	
N61	0.25378 (15)	0.01407 (14)	0.55669 (9)	0.0355 (3)	
C61	0.27566 (18)	−0.08185 (18)	0.56382 (11)	0.0386 (4)	
H61	0.356316	−0.078782	0.554042	0.046*	
C62	0.18226 (18)	−0.18381 (18)	0.58510 (12)	0.0391 (4)	
H62	0.198213	−0.251449	0.589358	0.047*	
C63	0.06403 (18)	−0.18932 (18)	0.60056 (11)	0.0375 (4)	
C64	0.04626 (18)	−0.08750 (18)	0.59427 (11)	0.0386 (4)	
H64	−0.032499	−0.087445	0.605725	0.046*	
C65	0.14146 (19)	0.01333 (18)	0.57162 (11)	0.0380 (4)	
H65	0.127867	0.082073	0.566563	0.046*	
C66	−0.0390 (2)	−0.3012 (2)	0.62263 (15)	0.0505 (5)	
H66A	−0.068265	−0.359616	0.578254	0.076*	
H66B	−0.109516	−0.285417	0.639936	0.076*	
H66C	−0.006565	−0.331754	0.664154	0.076*	

### Tris(4-methylpyridine N-oxide-κO)bis(thiocyanato-κN)cobalt(II) (2) Atomic displacement parameters (Å^2^)

**Table:**

	*U*^11^	*U*^22^	*U*^33^	*U*^12^	*U*^13^	*U*^23^
Co1	0.02863 (14)	0.02864 (14)	0.03621 (15)	0.01222 (11)	0.00333 (11)	0.00390 (11)
N1	0.0305 (8)	0.0337 (8)	0.0446 (9)	0.0128 (6)	0.0041 (6)	0.0059 (6)
C1	0.0335 (9)	0.0349 (9)	0.0376 (9)	0.0181 (8)	0.0016 (7)	0.0061 (7)
S1	0.0309 (2)	0.0339 (2)	0.0544 (3)	0.00922 (18)	0.00113 (19)	0.0089 (2)
N2	0.0308 (7)	0.0336 (8)	0.0355 (8)	0.0128 (6)	0.0040 (6)	0.0033 (6)
C2	0.0351 (9)	0.0358 (9)	0.0326 (9)	0.0186 (8)	0.0055 (7)	0.0031 (7)
S2	0.0334 (2)	0.0321 (2)	0.0426 (2)	0.00919 (17)	0.00627 (18)	0.00218 (18)
O11	0.0343 (6)	0.0293 (6)	0.0458 (7)	0.0120 (5)	0.0058 (5)	0.0012 (5)
N11	0.0318 (7)	0.0315 (7)	0.0372 (8)	0.0143 (6)	0.0035 (6)	0.0018 (6)
C11	0.0391 (9)	0.0367 (9)	0.0347 (9)	0.0184 (8)	0.0023 (7)	0.0063 (7)
C12	0.0365 (9)	0.0401 (10)	0.0336 (9)	0.0167 (8)	0.0000 (7)	0.0021 (7)
C13	0.0341 (9)	0.0349 (9)	0.0370 (9)	0.0166 (7)	0.0054 (7)	0.0037 (7)
C14	0.0370 (9)	0.0380 (9)	0.0351 (9)	0.0202 (8)	0.0042 (7)	0.0076 (7)
C15	0.0354 (9)	0.0408 (10)	0.0335 (9)	0.0198 (8)	0.0011 (7)	0.0030 (7)
C16	0.0413 (10)	0.0371 (10)	0.0482 (11)	0.0134 (8)	0.0023 (8)	0.0073 (8)
O21	0.0351 (7)	0.0347 (7)	0.0387 (7)	0.0074 (5)	0.0061 (5)	0.0018 (5)
N21	0.0335 (8)	0.0355 (8)	0.0328 (7)	0.0131 (6)	0.0049 (6)	0.0022 (6)
C21	0.0405 (10)	0.0393 (10)	0.0389 (10)	0.0211 (8)	0.0056 (8)	0.0062 (8)
C22	0.0350 (9)	0.0451 (11)	0.0418 (10)	0.0209 (8)	0.0027 (8)	0.0040 (8)
C23	0.0348 (9)	0.0402 (10)	0.0399 (10)	0.0172 (8)	0.0033 (7)	0.0012 (8)
C24	0.0372 (9)	0.0375 (9)	0.0405 (10)	0.0190 (8)	0.0054 (8)	0.0029 (8)
C25	0.0324 (9)	0.0406 (10)	0.0396 (10)	0.0184 (8)	0.0034 (7)	0.0038 (8)
C26	0.0358 (10)	0.0445 (11)	0.0670 (15)	0.0142 (9)	−0.0017 (10)	−0.0034 (10)
O31	0.0314 (6)	0.0334 (7)	0.0546 (8)	0.0116 (5)	0.0033 (6)	0.0134 (6)
N31	0.0310 (7)	0.0346 (8)	0.0424 (8)	0.0147 (6)	0.0029 (6)	0.0089 (6)
C31	0.0381 (10)	0.0429 (10)	0.0399 (10)	0.0222 (8)	0.0087 (8)	0.0069 (8)
C32	0.0448 (11)	0.0369 (10)	0.0430 (10)	0.0206 (8)	0.0061 (8)	0.0038 (8)
C33	0.0380 (10)	0.0375 (10)	0.0411 (10)	0.0158 (8)	0.0036 (8)	0.0077 (8)
C34	0.0407 (10)	0.0436 (10)	0.0368 (9)	0.0203 (8)	0.0061 (8)	0.0067 (8)
C35	0.0403 (10)	0.0386 (9)	0.0377 (9)	0.0207 (8)	0.0015 (8)	0.0019 (7)
C36	0.0555 (13)	0.0411 (11)	0.0532 (13)	0.0131 (10)	0.0079 (10)	0.0102 (9)
Co2	0.02840 (14)	0.03116 (15)	0.03437 (15)	0.01296 (12)	0.00080 (11)	0.00492 (11)
N3	0.0315 (8)	0.0350 (8)	0.0408 (8)	0.0132 (7)	−0.0009 (6)	0.0040 (6)
C3	0.0341 (9)	0.0363 (9)	0.0330 (9)	0.0186 (8)	0.0012 (7)	0.0021 (7)
S3	0.0320 (2)	0.0347 (2)	0.0445 (2)	0.00978 (17)	0.00310 (18)	−0.00029 (18)
N4	0.0303 (7)	0.0372 (8)	0.0359 (8)	0.0149 (6)	0.0018 (6)	0.0033 (6)
C4	0.0320 (9)	0.0371 (9)	0.0327 (9)	0.0171 (8)	0.0017 (7)	0.0049 (7)
S4	0.0360 (2)	0.0351 (2)	0.0443 (2)	0.00832 (18)	0.00144 (19)	0.00562 (19)
O41	0.0312 (6)	0.0328 (6)	0.0418 (7)	0.0119 (5)	0.0004 (5)	0.0077 (5)
N41	0.0310 (7)	0.0338 (8)	0.0358 (8)	0.0151 (6)	0.0001 (6)	0.0055 (6)
C41	0.0352 (9)	0.0416 (10)	0.0340 (9)	0.0219 (8)	0.0018 (7)	0.0038 (7)
C42	0.0381 (9)	0.0379 (9)	0.0368 (9)	0.0209 (8)	−0.0022 (7)	−0.0005 (7)
C43	0.0335 (9)	0.0359 (9)	0.0381 (9)	0.0164 (7)	−0.0017 (7)	0.0037 (7)
C44	0.0355 (9)	0.0382 (10)	0.0350 (9)	0.0160 (8)	0.0034 (7)	0.0040 (7)
C45	0.0376 (9)	0.0371 (9)	0.0327 (9)	0.0184 (8)	0.0014 (7)	0.0009 (7)
C46	0.0456 (11)	0.0375 (10)	0.0518 (12)	0.0129 (9)	0.0024 (9)	0.0023 (9)
O51	0.0306 (6)	0.0349 (7)	0.0607 (9)	0.0129 (5)	0.0022 (6)	−0.0025 (6)
N51	0.0306 (8)	0.0367 (8)	0.0462 (9)	0.0164 (6)	0.0036 (6)	0.0006 (7)
C51	0.0393 (10)	0.0420 (10)	0.0380 (10)	0.0219 (8)	0.0055 (8)	0.0071 (8)
C52	0.0406 (10)	0.0448 (10)	0.0332 (9)	0.0222 (8)	0.0004 (7)	0.0023 (8)
C53	0.0357 (9)	0.0384 (10)	0.0388 (10)	0.0165 (8)	0.0007 (7)	0.0017 (8)
C54	0.0395 (10)	0.0411 (10)	0.0425 (10)	0.0205 (8)	−0.0027 (8)	0.0046 (8)
C55	0.0373 (10)	0.0439 (11)	0.0432 (10)	0.0204 (8)	−0.0056 (8)	0.0002 (8)
C56	0.0575 (13)	0.0397 (11)	0.0535 (13)	0.0169 (10)	−0.0128 (10)	0.0027 (9)
O61	0.0366 (7)	0.0384 (7)	0.0380 (7)	0.0060 (6)	0.0000 (5)	0.0072 (6)
N61	0.0331 (8)	0.0383 (8)	0.0327 (7)	0.0127 (6)	0.0013 (6)	0.0054 (6)
C61	0.0314 (9)	0.0472 (11)	0.0416 (10)	0.0201 (8)	0.0037 (7)	0.0100 (8)
C62	0.0358 (10)	0.0427 (10)	0.0443 (10)	0.0209 (8)	0.0030 (8)	0.0104 (8)
C63	0.0331 (9)	0.0444 (10)	0.0359 (9)	0.0168 (8)	0.0028 (7)	0.0077 (8)
C64	0.0345 (9)	0.0484 (11)	0.0379 (10)	0.0221 (8)	0.0049 (7)	0.0043 (8)
C65	0.0388 (10)	0.0430 (10)	0.0360 (9)	0.0213 (8)	0.0016 (8)	0.0029 (8)
C66	0.0348 (10)	0.0507 (12)	0.0633 (14)	0.0140 (9)	0.0064 (9)	0.0161 (10)

### Tris(4-methylpyridine N-oxide-κO)bis(thiocyanato-κN)cobalt(II) (2) Geometric parameters (Å, º)

**Table:**

Co1—N1	2.0767 (16)	Co2—N3	2.0804 (16)
Co1—N2	2.0895 (15)	Co2—N4	2.0844 (16)
Co1—O11	1.9949 (13)	Co2—O41	1.9999 (13)
Co1—O21	1.9989 (14)	Co2—O51	1.9880 (14)
Co1—O31	2.0045 (14)	Co2—O61	1.9941 (14)
N1—C1	1.162 (2)	N3—C3	1.160 (2)
C1—S1	1.6362 (19)	C3—S3	1.6388 (19)
N2—C2	1.170 (2)	N4—C4	1.164 (2)
C2—S2	1.6352 (19)	C4—S4	1.6353 (19)
O11—N11	1.348 (2)	O41—N41	1.3494 (19)
N11—C11	1.348 (2)	N41—C41	1.348 (2)
N11—C15	1.348 (2)	N41—C45	1.345 (2)
C11—H11	0.9500	C41—H41	0.9500
C11—C12	1.374 (3)	C41—C42	1.372 (3)
C12—H12	0.9500	C42—H42	0.9500
C12—C13	1.396 (3)	C42—C43	1.397 (3)
C13—C14	1.388 (3)	C43—C44	1.392 (3)
C13—C16	1.499 (3)	C43—C46	1.500 (3)
C14—H14	0.9500	C44—H44	0.9500
C14—C15	1.372 (3)	C44—C45	1.375 (3)
C15—H15	0.9500	C45—H45	0.9500
C16—H16A	0.9800	C46—H46A	0.9800
C16—H16B	0.9800	C46—H46B	0.9800
C16—H16C	0.9800	C46—H46C	0.9800
O21—N21	1.349 (2)	O51—N51	1.351 (2)
N21—C21	1.348 (2)	N51—C51	1.346 (3)
N21—C25	1.345 (3)	N51—C55	1.344 (3)
C21—H21	0.9500	C51—H51	0.9500
C21—C22	1.376 (3)	C51—C52	1.373 (3)
C22—H22	0.9500	C52—H52	0.9500
C22—C23	1.389 (3)	C52—C53	1.395 (3)
C23—C24	1.394 (3)	C53—C54	1.385 (3)
C23—C26	1.496 (3)	C53—C56	1.495 (3)
C24—H24	0.9500	C54—H54	0.9500
C24—C25	1.367 (3)	C54—C55	1.369 (3)
C25—H25	0.9500	C55—H55	0.9500
C26—H26A	0.9800	C56—H56A	0.9800
C26—H26B	0.9800	C56—H56B	0.9800
C26—H26C	0.9800	C56—H56C	0.9800
O31—N31	1.346 (2)	O61—N61	1.349 (2)
N31—C31	1.352 (3)	N61—C61	1.345 (3)
N31—C35	1.348 (3)	N61—C65	1.345 (2)
C31—H31	0.9500	C61—H61	0.9500
C31—C32	1.371 (3)	C61—C62	1.368 (3)
C32—H32	0.9500	C62—H62	0.9500
C32—C33	1.397 (3)	C62—C63	1.392 (3)
C33—C34	1.392 (3)	C63—C64	1.389 (3)
C33—C36	1.497 (3)	C63—C66	1.495 (3)
C34—H34	0.9500	C64—H64	0.9500
C34—C35	1.373 (3)	C64—C65	1.377 (3)
C35—H35	0.9500	C65—H65	0.9500
C36—H36A	0.9800	C66—H66A	0.9800
C36—H36B	0.9800	C66—H66B	0.9800
C36—H36C	0.9800	C66—H66C	0.9800
			
N1—Co1—N2	179.11 (7)	N3—Co2—N4	178.20 (6)
O11—Co1—N1	93.86 (6)	O41—Co2—N3	93.31 (6)
O11—Co1—N2	86.87 (6)	O41—Co2—N4	88.08 (6)
O11—Co1—O21	122.93 (6)	O51—Co2—N3	86.15 (6)
O11—Co1—O31	121.98 (6)	O51—Co2—N4	92.14 (6)
O21—Co1—N1	87.05 (6)	O51—Co2—O41	122.04 (6)
O21—Co1—N2	92.13 (6)	O51—Co2—O61	115.95 (6)
O21—Co1—O31	115.07 (6)	O61—Co2—N3	87.50 (6)
O31—Co1—N1	87.43 (6)	O61—Co2—N4	92.75 (6)
O31—Co1—N2	92.62 (6)	O61—Co2—O41	121.94 (6)
Co1—N1—Co	167.85 (16)	Co2—N3—Co3	168.81 (15)
N1—C1—S1	179.28 (19)	N3—C3—S3	179.8 (2)
C2—N2—Co1	164.31 (15)	C4—N4—Co2	161.93 (15)
N2—C2—S2	179.49 (19)	N4—C4—S4	179.57 (19)
N11—O11—Co1	117.79 (10)	N41—O41—Co2	117.09 (10)
C11—N11—O11	119.39 (15)	C41—N41—O41	119.69 (16)
C11—N11—C15	121.18 (16)	C45—N41—O41	119.27 (15)
C15—N11—O11	119.44 (15)	C45—N41—C41	121.03 (16)
N11—C11—H11	120.0	N41—C41—H41	119.9
N11—C11—C12	120.10 (17)	N41—C41—C42	120.12 (18)
C12—C11—H11	120.0	C42—C41—H41	119.9
C11—C12—H12	119.7	C41—C42—H42	119.5
C11—C12—C13	120.61 (18)	C41—C42—C43	120.98 (18)
C13—C12—H12	119.7	C43—C42—H42	119.5
C12—C13—C16	121.34 (18)	C42—C43—C46	121.69 (18)
C14—C13—C12	117.13 (17)	C44—C43—C42	116.75 (18)
C14—C13—C16	121.49 (17)	C44—C43—C46	121.53 (18)
C13—C14—H14	119.4	C43—C44—H44	119.5
C15—C14—C13	121.15 (17)	C45—C44—C43	120.98 (18)
C15—C14—H14	119.4	C45—C44—H44	119.5
N11—C15—C14	119.83 (17)	N41—C45—C44	120.12 (17)
N11—C15—H15	120.1	N41—C45—H45	119.9
C14—C15—H15	120.1	C44—C45—H45	119.9
C13—C16—H16A	109.5	C43—C46—H46A	109.5
C13—C16—H16B	109.5	C43—C46—H46B	109.5
C13—C16—H16C	109.5	C43—C46—H46C	109.5
H16A—C16—H16B	109.5	H46A—C46—H46B	109.5
H16A—C16—H16C	109.5	H46A—C46—H46C	109.5
H16B—C16—H16C	109.5	H46B—C46—H46C	109.5
N21—O21—Co1	116.67 (10)	N51—O51—Co2	120.20 (11)
C21—N21—O21	119.83 (16)	C51—N51—O51	120.45 (17)
C25—N21—O21	118.85 (16)	C55—N51—O51	118.22 (17)
C25—N21—C21	121.27 (17)	C55—N51—C51	121.32 (17)
N21—C21—H21	120.0	N51—C51—H51	120.0
N21—C21—C22	119.92 (18)	N51—C51—C52	120.03 (18)
C22—C21—H21	120.0	C52—C51—H51	120.0
C21—C22—H22	119.8	C51—C52—H52	119.7
C21—C22—C23	120.48 (18)	C51—C52—C53	120.55 (18)
C23—C22—H22	119.8	C53—C52—H52	119.7
C22—C23—C24	117.52 (18)	C52—C53—C56	122.25 (18)
C22—C23—C26	121.97 (19)	C54—C53—C52	117.00 (18)
C24—C23—C26	120.49 (19)	C54—C53—C56	120.74 (18)
C23—C24—H24	119.7	C53—C54—H54	119.3
C25—C24—C23	120.64 (18)	C55—C54—C53	121.34 (19)
C25—C24—H24	119.7	C55—C54—H54	119.3
N21—C25—C24	120.14 (18)	N51—C55—C54	119.73 (18)
N21—C25—H25	119.9	N51—C55—H55	120.1
C24—C25—H25	119.9	C54—C55—H55	120.1
C23—C26—H26A	109.5	C53—C56—H56A	109.5
C23—C26—H26B	109.5	C53—C56—H56B	109.5
C23—C26—H26C	109.5	C53—C56—H56C	109.5
H26A—C26—H26B	109.5	H56A—C56—H56B	109.5
H26A—C26—H26C	109.5	H56A—C56—H56C	109.5
H26B—C26—H26C	109.5	H56B—C56—H56C	109.5
N31—O31—Co1	116.49 (10)	N61—O61—Co2	117.27 (11)
O31—N31—C31	118.85 (16)	C61—N61—O61	118.81 (16)
O31—N31—C35	119.84 (16)	C61—N61—C65	121.31 (17)
C35—N31—C31	121.29 (17)	C65—N61—O61	119.85 (16)
N31—C31—H31	120.1	N61—C61—H61	119.9
N31—C31—C32	119.83 (18)	N61—C61—C62	120.28 (18)
C32—C31—H31	120.1	C62—C61—H61	119.9
C31—C32—H32	119.5	C61—C62—H62	119.7
C31—C32—C33	120.92 (19)	C61—C62—C63	120.57 (18)
C33—C32—H32	119.5	C63—C62—H62	119.7
C32—C33—C36	121.11 (19)	C62—C63—C66	120.74 (19)
C34—C33—C32	116.96 (18)	C64—C63—C62	117.33 (18)
C34—C33—C36	121.90 (19)	C64—C63—C66	121.93 (18)
C33—C34—H34	119.5	C63—C64—H64	119.6
C35—C34—C33	121.01 (19)	C65—C64—C63	120.77 (18)
C35—C34—H34	119.5	C65—C64—H64	119.6
N31—C35—C34	119.84 (18)	N61—C65—C64	119.70 (18)
N31—C35—H35	120.1	N61—C65—H65	120.1
C34—C35—H35	120.1	C64—C65—H65	120.1
C33—C36—H36A	109.5	C63—C66—H66A	109.5
C33—C36—H36B	109.5	C63—C66—H66B	109.5
C33—C36—H36C	109.5	C63—C66—H66C	109.5
H36A—C36—H36B	109.5	H66A—C66—H66B	109.5
H36A—C36—H36C	109.5	H66A—C66—H66C	109.5
H36B—C36—H36C	109.5	H66B—C66—H66C	109.5

### Tris(4-methylpyridine N-oxide-κO)bis(thiocyanato-κN)cobalt(II) (2) Hydrogen-bond geometry (Å, º)

**Table:**

*D*—H···*A*	*D*—H	H···*A*	*D*···*A*	*D*—H···*A*
C11—H11···S4^i^	0.95	2.88	3.8088 (19)	166
C21—H21···S2^ii^	0.95	2.87	3.739 (2)	153
C24—H24···S1^iii^	0.95	2.90	3.841 (2)	169
C25—H25···O31^iii^	0.95	2.63	3.259 (2)	124
C31—H31···O21^iii^	0.95	2.44	3.277 (2)	146
C34—H34···S3	0.95	2.99	3.754 (2)	138
C41—H41···S3^iv^	0.95	2.97	3.6966 (19)	134
C45—H45···S2	0.95	2.89	3.6082 (19)	133
C55—H55···O61^v^	0.95	2.46	3.184 (2)	133
C61—H61···O51^v^	0.95	2.48	3.233 (2)	136
C62—H62···S3^v^	0.95	2.98	3.900 (2)	162
C64—H64···N4^vi^	0.95	2.64	3.484 (2)	148
C65—H65···S4^vi^	0.95	3.00	3.870 (2)	154

Symmetry codes: (i) *x*+1, *y*+1, *z*; (ii) −*x*+1, −*y*+1, −*z*; (iii) −*x*+2, −*y*+1, −*z*; (iv) −*x*+1, −*y*+1, −*z*+1; (v) −*x*+1, −*y*, −*z*+1; (vi) −*x*, −*y*, −*z*+1.
