# Supplementary material for: Synthesis and crystal structure of diiso­thio­cyanato­tetra­kis­(4-methyl­pyridine N-oxide)cobalt(II) and diiso­thio­cyanato­tris­(4-methyl­pyridine N-oxide)cobalt(II) showing two different metal coordination polyhedra
Source: Acta Crystallogr E Crystallogr Commun. 2024 Jan 26;80(Pt 2):174–9. doi: 10.1107/S2056989024000471 (PMC10848974; doi:10.1107/S2056989024000471)
Supplement: Supplementary file 4 [file e-80-00174-sup4.pdf]

Acta Cryst E STRUCTURE REPORTS

Volume 80 (2024)

Supporting information for article:

Synthesis and crystal structure of diisothiocyanatotetrakis(4-methylpyridine *N*-oxide)cobalt(II) and diisothiocyanatotris(4-methylpyridine *N*-oxide)cobalt(II) showing two different metal coordination polyhedra

Christian Näther and Inke Jess

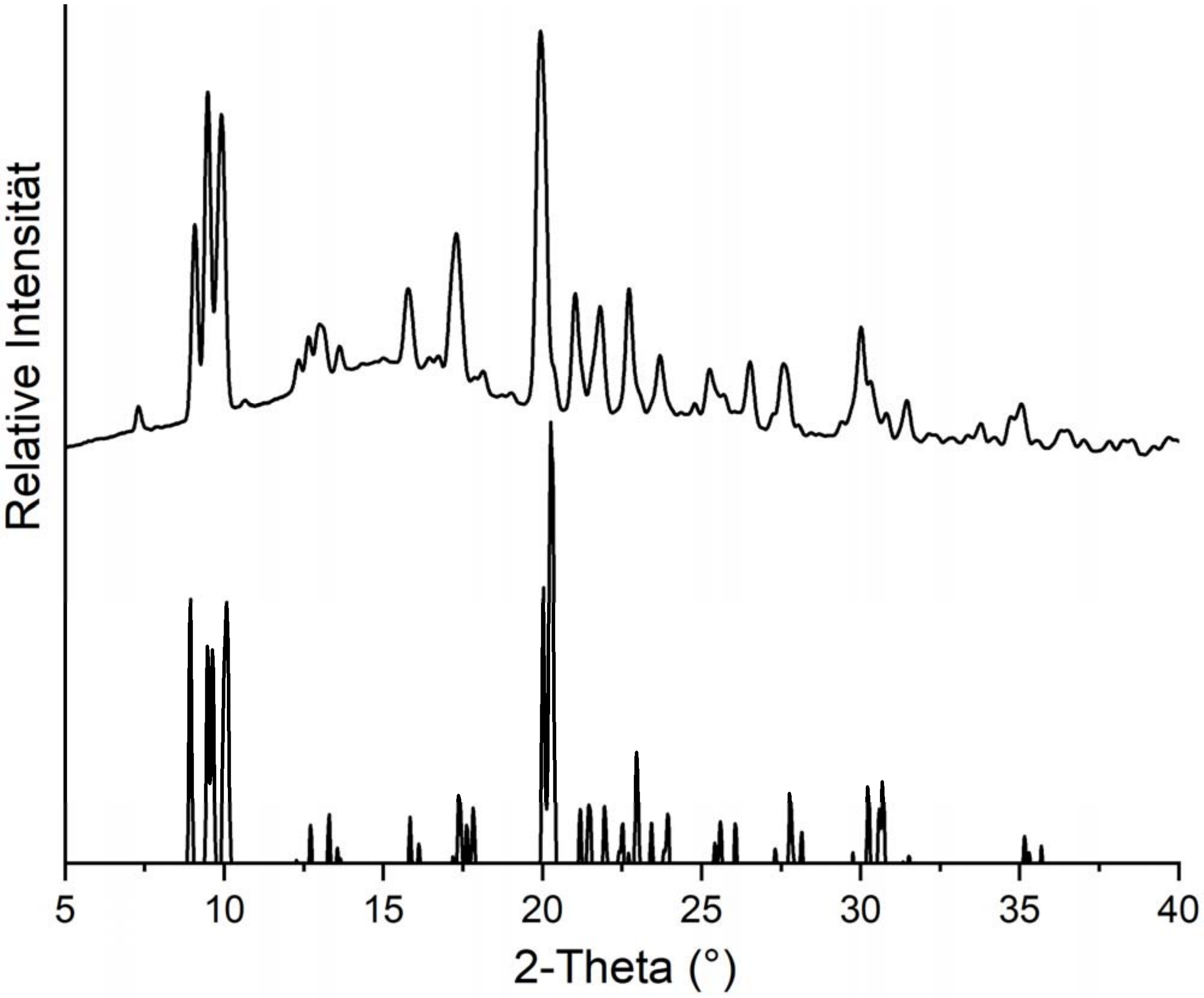

Fig. S1.

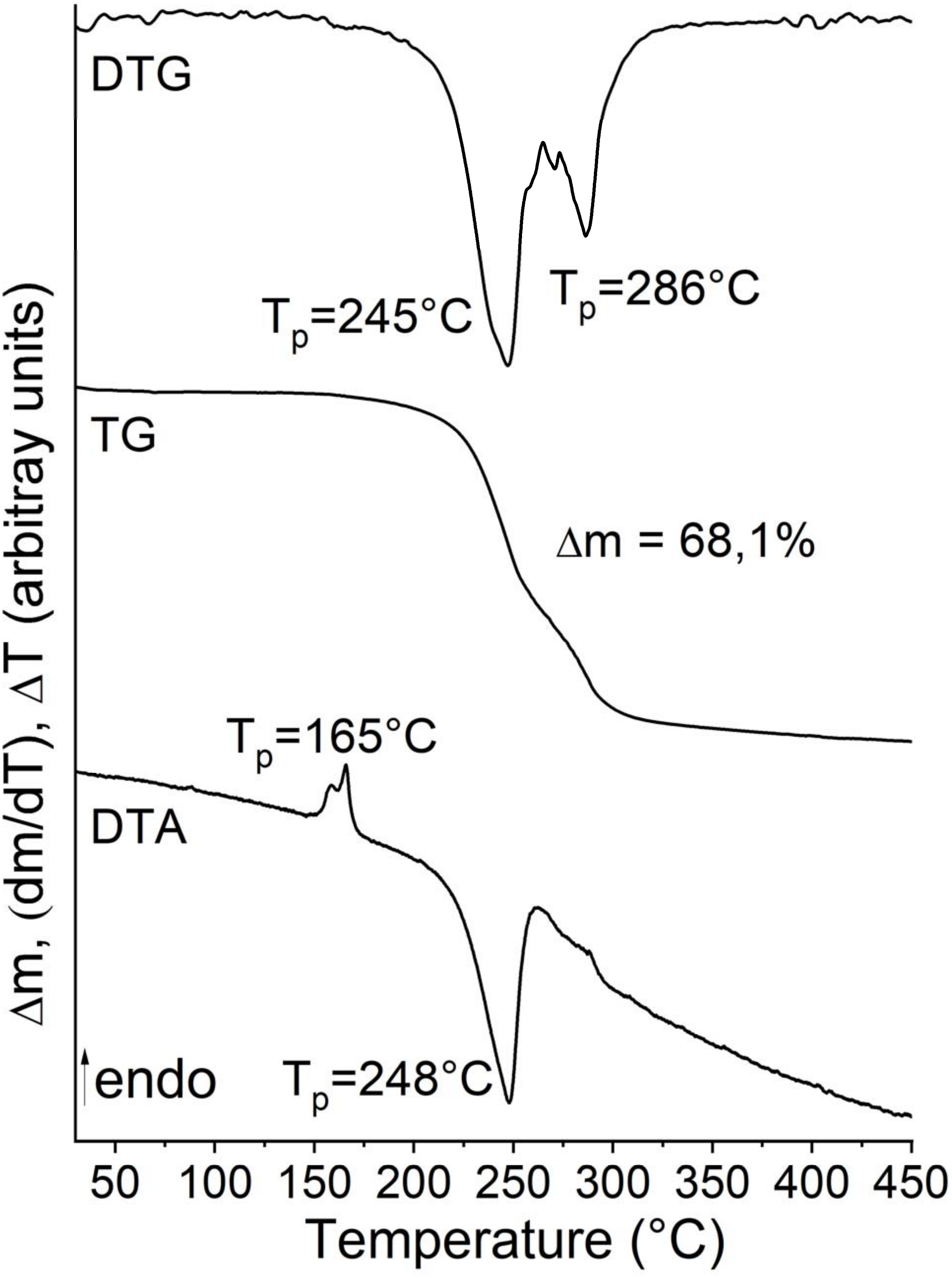
DTG
T_p=245°C
T_p=286°C
TG
Δm = 68,1%
T_p=165°C
DTA
endo
T_p=248°C
Δm, (dm/dT), ΔT (arbitray units)
50
100
150
200
250
300
350
400
450
Temperature (°C)

Fig. S2

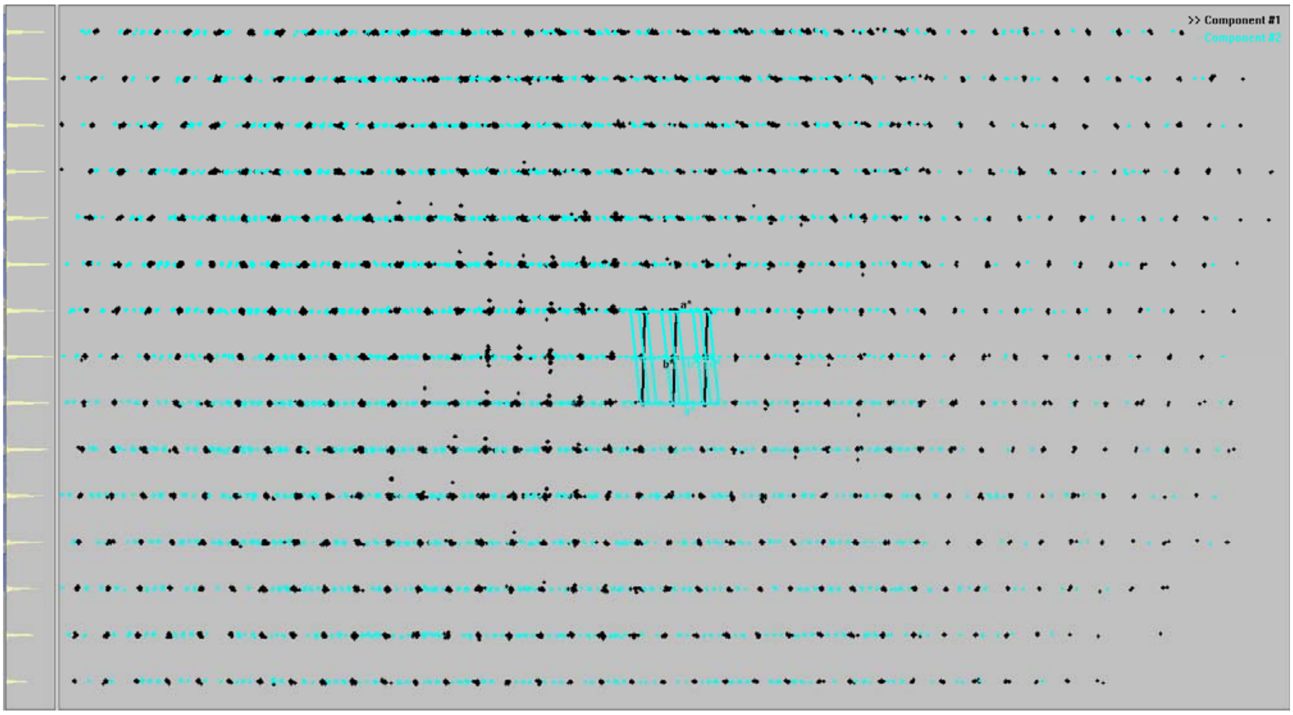

Fig. S3
